# Self-generated Local Heating Induced Nanojoining for Room Temperature Pressureless Flexible Electronic Packaging

**DOI:** 10.1038/srep09282

**Published:** 2015-03-19

**Authors:** Peng Peng, Anming Hu, Adrian P. Gerlich, Yangai Liu, Y. Norman Zhou

**Affiliations:** 1 Centre for Advanced Materials Joining, University of Waterloo, 200 University Avenue West, Waterloo, ON, N2L 3G1, Canada; 2 Waterloo Institute for Nanotechnology, University of Waterloo, 200 University Avenue West, Waterloo, ON, N2L 3G1, Canada; 3 Mechanical, Aerospace and Biomedical Engineering Department, University of Tennessee, 1512 Middle Drive, Knoxville, TN 37996-2210, US; 4 School of Materials Science and Technology, China University of Geosciences (Beijing), Beijing 100083, China

## Abstract

Metallic bonding at an interface is determined by the application of heat and/or pressure. The means by which these are applied are the most critical for joining nanoscale structures. The present study considers the feasibility of room-temperature pressureless joining of copper wires using water-based silver nanowire paste. A novel mechanism of self-generated local heating within the silver nanowire paste and copper substrate system promotes the joining of silver-to-silver and silver-to-copper without any external energy input. The localized heat energy was delivered in-situ to the interfaces to promote atomic diffusion and metallic bond formation with the bulk component temperature stays near room-temperature. This local heating effect has been detected experimentally and confirmed by calculation. The joints formed at room-temperature without pressure achieve a tensile strength of 5.7 MPa and exhibit ultra-low resistivity in the range of 101.3 nOhm·m. The good conductivity of the joint is attributed to the removal of organic compounds in the paste and metallic bonding of silver-to-copper and silver-to-silver. The water-based silver nanowire paste filler material is successfully applied to various flexible substrates for room temperature bonding. The use of chemically generated local heating may become a potential method for energy in-situ delivery at micro/nanoscale.

The atomic level interconnection of components to achieve metallurgical bonds is a key requirement for micro/nano-electronic devices since conductive joints are needed in transistors^1^,^2^, sensors^3^, and solar cells^4^ *etc*. Conventionally, external energy inputs are needed to accelerate diffusion and mixing of atoms at the interfaces for metallic bond formation. These excitation energies are generally applied through external sources during soldering & sintering^5^,^6^, laser welding^7^,^8^, resistance welding^9^, friction welding^10^,^11^, microwave sintering^12^, pressure welding^13^, and delivered to the interfaces where connections are required. Generally, the interfaces are mostly in bulk size and the energy requirements are large in these conventional joining processes. Thermite welding is one category where heat is generated in-situ by an exothermic reaction, however the process results in high temperatures which also melt the bulk material. When the device and joint size reduce to micro- and/or nano-scale, the energy delivery should be more precise, efficient and controllable. Meanwhile, some drawbacks will arise if the energy is still delivered to the bulk surrounding material. For example, in the case of thermal heating, a typical lead-free soldering process reaches 200 to 300°C to obtain conductive joints^6^,^14^, which severely restricts soldering for heat-sensitive components in electronic devices, such as flexible electronic paper and organic light-emitting devices. Regarding laser sources, a beam diameter below 1 μm is difficult to achieve, and energy density exponentially increases with decreasing beam size. Therefore, targeted energy delivery routes should be developed to better control energies in a localized region without affecting surroundings for micro/nano-electronic fabrication or flexible electronic device assembly.

Only a few new methods for delivering energy at the nanoscale for welding applications have been reported, such as nanoscale soldering of individual gold nanowires by electrical welding using metal solder^15^, and light-induced plasmonic local heating to achieve nanojunctions of silver nanowires^16^. The former directly adopted Joule heating in a nanosized region while the latter concentrated photon energy onto a nanosized “hot-spot” region with the help of plasmonic effects to generate heat locally. Similarly in these two methods, only a small amount of energy was delivered and focused onto nanoscale areas to induce local melting and then facilitate nanojoining. It is worth noting that one of the benefits at the nanoscale occurs in diffusion bonding of metallic nanomaterials, allowing joining at relatively low temperatures or even room-temperature due to the increased surface energy, sintering pressure and diffusion rates^17^ when particle size is reduced <100 nm. Thus, the energy requirement for solid state joining at the nanoscale is very limited. Even cold welding without external energy input has been demonstrated by contacting two gold or silver nanowires, where surface-atom diffusion and self-orientation attachment promotes nanojoining^18^,^19^.

To date, low temperature interconnection processes using noble metal nanoparticle pastes appear to be a promising alternative for lead-free electronic packaging and flexible electronics interconnections. Room-temperature sintering of silver nanoparticles has also been realized through removing the capping agent on nanoparticle surface or the dispersant in paste with organic solvent or ion solutions^20^,^21^. However, the utilization of solvents or other solutions during the joining process may introduce containments, increase the cost and thereby restrict their practical applications. On the contrary, by removing the organics in the paste, clean nanopastes could simplify the joining significantly.

In the present work water-based silver nanowire pastes are utilized to bond two copper substrates with excellent conductivity at room temperature without solvents. The mechanism of room-temperature joining using water-based nanowire paste is studied in terms of an innovative localized heat generation. It was found that self-generated local heating effects and in-situ energy release at the interfaces within the silver nanowire paste and copper system contribute to the joining process when the ambient temperature is held near room-temperature. Here, the origin of such local heating and its contributions to the metallic bond formation at silver-silver and silver-copper interfaces are explored for the first time. The effect of local heating generation is investigated during room-temperature pressureless bonding which can be applied on various organic substrates for flexible electronic packaging applications.

## Results and Discussion

**Figure 1:**
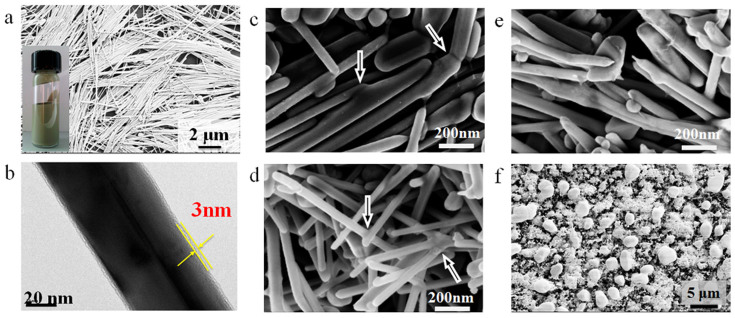
(a) SEM image of silver nanowire paste (inset: optical image of highly concentrated Ag NW paste); (b) TEM image of single Ag NW with 3 nm organic protected layer on the surface. Microstructures of sintered Ag NW paste at (c) 200°C for 5 min on Si substrates; (d) Room-temperature, (e) 200°C for 1 hr and (f) 300°C for 1 hr bonded Ag NW on Cu substrates.

**Figure 2:**
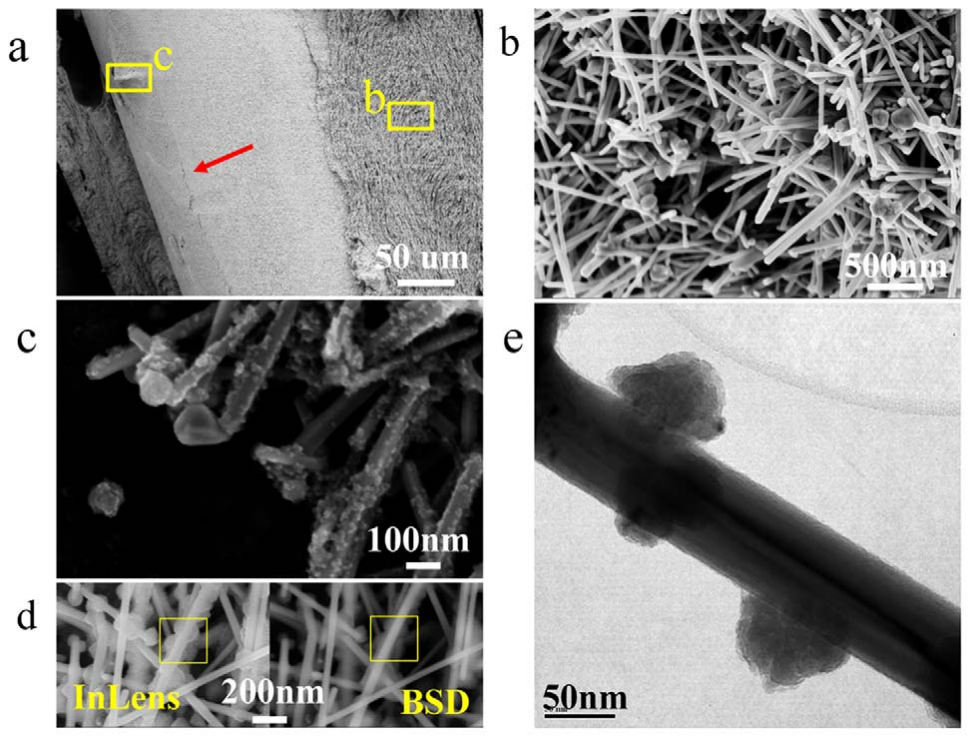
SEM images of (a) fractured sample and (b) fractured surface on Ag NW side. (c) Nanoparticle decorated Ag NW layer. (d) Comparison of InLens and Back-scatting images of NP decorated NWs, indicating the associated NPs have different composites from Ag NWs as highlighted in boxes. (e) TEM micrograph of NP on the surface of Ag NW taken from Cu-Ag interface of bonded sample.

Figure 1 shows the microstructure of Ag NWs. These Ag NWs synthesized from polyol solution were about 15 μm long and 100–200 nm thick on average. On the surface of the Ag NW, a thin amorphous layer around 3 nm was observed, see Figure 1b, which is PVP organic compound adsorbed on the surface during the growth process which prevented oxidation of the Ag NWs. With the help of these organics, the coated Ag NWs are stable and the paste could be stored for more than 8 months without any degradation of performance as filler materials for low temperature bonding. Though Ag NWs could be separated and agglomerated from the paste, ultrasonication can also be employed to re-disperse them easily and use for bonding. To investigate the sintering behavior, these NW pastes were heated on silicon wafers and copper foils in air, so that the two different substrates could be compared from the standpoint of their different chemical inertness and activity with the water-based Ag NW paste. On the Si wafer, no significant joining of Ag NWs was observed at temperatures below than 200°C. Ag NWs show no changes after heating at 100°C for 1 hour, while they started to connect to their nearest neighbors after sintering for 5 mins at 200°C (as indicated by arrows in Figure 1c). After heating at 200°C for 1 hour, some NWs became thinner and even broken due to long-range solid state diffusion and growth. However, the Ag NW paste on a Cu substrate was found to sinter at room temperature with some wires interconnected to each other and formed cross-wire or tri-junction structures as shown in Figure 1d. The paste sample on Cu sintered at 200°C for 1 hour (Figure 1e) shows no significant difference with that on Si. If the temperature increases to 300°C, most of the Ag NWs were broken into pieces and bundled with others to form particles as illustrated in Figure 1f. The Cu substrate exhibited a higher activity than Si to promote sintering of Ag NWs and lowers the sintering temperature down to room-temperature, suggesting Ag NW pastes have a potential to bond with Cu based substrates at low temperatures. To confirm this, two 250 μm Cu wires were bonded using the Ag NW paste at room-temperature (see inset image of Figure S1a). The cross-section shows that the Cu and Ag NW were in close contact on the round interface, without obvious unbounded interfaces. Due to the nature of liquid in the Ag NW paste, it can flow around the Cu wire and fill the gap between two components to make a complete connection around the circumference. SEM analysis with an EDX line scan across the interface indicates only Cu and Ag with undetectable oxygen (see Figure S1b). On the fracture surface, a thin Ag NW layer on Cu wire surface was observed as shown in Figure 2a. The Ag NWs were interconnected at room-temperature in the filler Ag NWs (Figure 2b, c) similar to the previous sintering results indicate in Figure 1d. Meanwhile, many nanoparticles were clearly observed on the surface of Ag NW within this thin layer (Figure 2c) close to the Ag-Cu interface. These nanoparticles show darker contrast than Ag NWs in a backscattered electron image compared to an in-lens image as indicated in Figure 2d, suggesting that they contain lighter elements compared with Ag, in this case Cu. Figure 2e clearly illustrates the nanoparticles grew on the surface of Ag NWs. According to EDX analysis via HRTEM and micro-XRD, these nanoparticles have been identified as CuO (see Supplementary Note 1 and Figure S2). The difference in chemical potentials of Cu and Ag implies that Cu would be oxidized to Cu ions and migrate to defect areas on the surface of the Ag NWs, and combine with oxygen to grow Cu_2_O which later transforms to CuO^22^,^23^.

**Figure 3:**
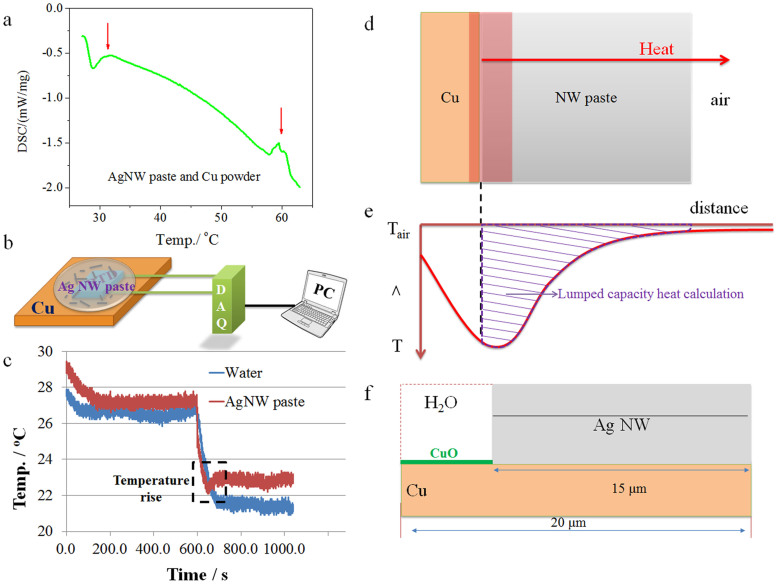
(a) DSC curve of Ag NW paste with Cu powder heating in air. (b) Schemes of temperature testing configurations of bare Cu substrate and Cu-Ag NW paste system at room temperature. (c) Temperature profiles of pure water and Ag NW paste with Cu substrate. (d) Cu and NW paste plate assembly for heat energy calculation using lumped capacity model and (e) its schematic of temperature profile with calculated localized heat energies at the nanoscale. (f) Side view of localized interface with size configurations.

To see if there is any self-generated heating which promoted joining of Ag NWs at room-temperature in the Cu-Ag NW system, the Ag NW paste and Cu powder was mixed and immediately transferred into a crucible to measure the temperature change from room-temperature to 60°C using DSC. The results in Figure 3a show two exothermal peaks located at round 32 and 60°C, respectively. The first one might be due to the oxidation of Cu and second one may be associated with the surface area reduction during joining of Cu to Ag, or Ag to Ag. A further assessment of this possible heating was done by monitoring the temperature change using resistance temperature detector (RTD) once the Ag NW paste was dropped onto a Cu substrate as configured in Figure 3b. The temperature profiles of water-Cu compared to NW paste-Cu systems are shown in Figure 3c. After dropping water or water-based paste onto the substrates, the temperatures all decreased because of heat absorption and evaporation of water and finally reached a constant temperature at around 22°C. However, the temperature history in the sample containing the Ag NW paste shows a peak feature at the end of decreasing tail, highlighted in the dashed box of Figure 3c. This slight increase in temperature is evidence that heat is generated within the Cu and Ag NW paste system, which represents the local heating, measurable in the macro-scale system. The local heating would dissipate through various heat sinks in the Cu - Ag NW paste system, including heat transfer to surroundings through the Cu substrate, paste and the evaporation of water. Although the measurable temperature difference is only a 0.5–1°C reading from the curve, the localized thermal energy per volume is likely to be much higher since it is generated at the nanosized interfaces and transferred to macro components. The experiments show that this local heating would be sufficient to promote joining of Ag NWs and Ag NWs to Cu substrates. However, it only behaves as a slight temperature increase, and thus does not affect the heat sensitive substrate during bonding process, which makes it a very promising approach for room-temperature bonding.

To calculate this local heating energy, the lumped capacity model^24^ was used at the interfaces based on measured temperatures. In this model, the paste and copper are two parallel contacted plates and the temperature difference inside each plate is negligible, see configurations in Figure 3d. Since the heat generations mostly occurred in the NW paste side close to interfaces and the temperature profile of Cu substrate was not measured, the heat energy is only calculated on NW paste side. The evaporation of water and absorption by components of system were not considered. Thus, according to Newton's Law of Cooling, the rate of heat loss of the plates (NW paste) is proportional to the temperature difference between them and their surroundings. Thus, the first-order differential equation is,
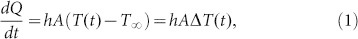
where *Q* is the thermal energy; *h* is the heat transfer coefficient between the plate and air, typically *h* for air is approximately *1–2 Wm^−2^K^−1^* for free convection^25^. *A* is the area across the interfaces of the heat being transferred, *T* is the temperature, a function of time *t* in the paste plate; *T_∞_* is the temperature of surrounding, here we take *T_∞_* = *T_air_*. The time-dependent thermal gradient Δ*T*(*t*) corresponds to the difference *T*(*t*) − *T_air_*, between the plate and air.
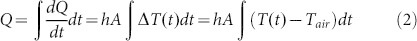

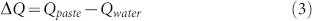

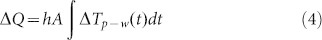
Therefore, the transferred heat energy *Q* is calculated by integrating *dQ/dt* as indicated in Eq. 2. In this case, the local heat *ΔQ* is the difference between the Cu - NW paste and Cu - water system (here Cu - water system is a reference for calculating how much heat generated due to the application of the NW paste), which can be calculated as shown in Eq. (3–4) by integrating the area of two temperature profiles (Figure 3c) and taking the difference of them, 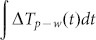. In this lumped capacity model, the heat sink on the Cu plate side was not calculated. Therefore, this calculation only captured a portion of the generated heat as schematically indicated in Figure 3e (shaded area). Here, we only consider a nanosized interface: one single nanowire flat on Cu surface. The width of one side of the Ag NW is 100 nm (which is the side length of a NW with an overall thickness of 162 nm due to the pentagon shape of its cross section); and the length of the Ag NW is 15 μm, while the length of the Cu surface is 20 μm since the Cu surface was not fully covered as shown in Figure 3f. This coverage value corresponds to 46% in this case, which is close to the range of 40–50% based on TEM images. Therefore, *A = 162 nm × 20 μm* is the projection area of the single NW - Cu nano-interface system on paste-air interface (based on the parallel plate assumption). The minimum heat transfer coefficient of still air at room temperature was assumed to be *1 Wm^−2^K^−1^* to calculate the minimum transferred heat energy. The difference between the two curves is around *433 K·s* (from the peak temperature after applying paste/water at *730 s* to the stable temperatures at *1038 s*). Then, the local heat *ΔQ* will be *1.40 nJ* (on the NW paste side for a thin slice with cross section area of *A*).

**Figure 4:**
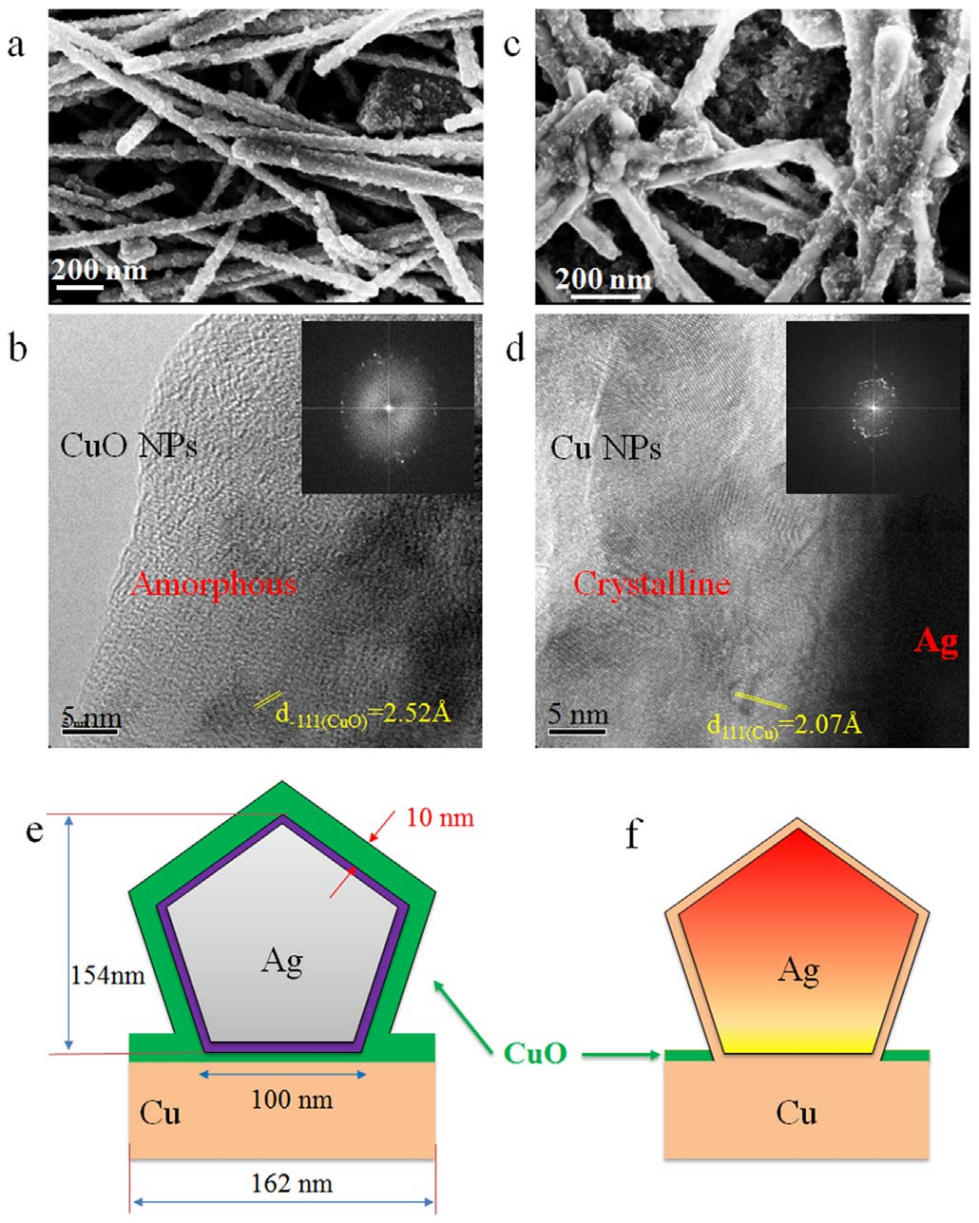
SEM and TEM images of (a, b) CuO grown on the surface of Ag NW at room-temperature with large portions of amorphous structures and (c, d) increasing Cu nanocrystalline structures after heating at 80°C for 3 hrs; insets are FFT images. Cross-section view of simplified Cu-Ag localized interface (e) before and (f) after temperature rise due to CuO-PVP reaction.

The fracture surfaces of samples bonded at room-temperature without further heat treatment and heating at higher temperatures were observed. At room-temperature, most of these CuO nanoparticles grown on the NW surface, see Figure 4a (also shown in Figure S3a). They exhibit an amorphous-like structure as the TEM and inset FFT images indicated in Figure 4b although a very few of nanoparticles illustrate the (−111) plane of CuO. However, CuO nanoparticles decreased significantly after heating at 100°C (Figure S3b). The reduction of oxygen content based on EDX analysis also supports this observation. After treating at 150°C, CuO nanoparticles were barely observed (as shown in Figure S3c, d). In a previous report^22^, the residual PVP was shown to serve as reducing agent to react with CuO and obtain Cu nanomaterials, and this has been employed for noble metal nanomaterials synthesis^26^,^27^,^28^. To test this hypothesis, the CuO-Ag NW composite was heated in water solutions containing PVP at a higher temperature of 80°C as studied by Xiong *et al.*^27^, which confirmed the reducing effect of PVP. After heating, the CuO nanoparticles became much smaller and more agglomerations were observed in Figure 4c. HRTEM imaging indicates that the amorphous structures decreased and more crystalline nanoparticles were observed after reaction (see the HRTEM and FFT images in Figure 4d). Based on lattice diffraction and EDX results, most of the CuO nanoparticles on the surface of the Ag NWs were found to have transformed to Cu nanocrystals (details are shown in Supplementary Note 2 and Figure S4). This suggested that at higher temperatures PVP on Ag NW surfaces could clean the CuO formed during the bonding process at room-temperature. It can reduce or prevent the oxidation of Cu and Ag during bonding process because of local heating as previously discussed. This “self-cleaning” mechanism among Cu-PVPs-Ag system at low temperature has been discussed in detail in a previous report^22^.

It is worth noting that the local heating may originate from the oxidation of copper and the reaction of PVP on the Cu-Ag interfaces, the reduction of surface during joining of Ag-Ag or Cu-Ag could contribute to the generation of heat as well. To understand mechanism of this self-generated local heating induced nanojoining at the nanoscale, some simple calculations of the temperature rise can be made based on the enthalpy of reactions. First, the oxidation of Cu will release heat *ΔH_1_* as shown in Eq. 5. Due to the long term oxidation of Cu during the bonding time scale of a few seconds to minutes, the heat energy is difficult to calculate because of a heat sink effect. However, this reaction will produce CuO and supply the initial heat for CuO reacting with PVP, which could generate heat *ΔH_2_* (Eq. 6). Meanwhile, the removal of PVP at the interface and the generated heat can promote the interdiffusion of Ag and Cu to form metallic bonds, see Figure 4e–f. This reaction is shown in Eq. 7 and *ΔH_3_* is the released heat calculated according to reduced surfaces of Cu and Ag because the composition of *Ag_x_Cu* and its reaction heat is difficult to quantify. Here, we simply consider both of the surfaces of Cu and Ag are the (100) plane and surface energies of them can be found in literature^29^. The heats from the enthalpy change of the first two reactions Eq. 5–6 can be obtained from thermochemical data handbooks^30^. Since the thermodynamic data of PVP is difficult to obtain, one can simplify the long chain *R* as methyl.
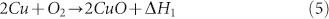

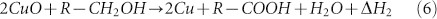

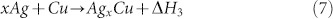


Due to a layer of CuO coated on Cu and Ag surface in the single NW-Cu localized system and the CuO-PVP reaction mainly occurring on the Ag NW surface, the heat *ΔH_2_* will directly conduct to Ag NW to heat it up. It is difficult for this heat to transfer to the surrounding water/air because of their small thermal conductivities, *K_H2O_ = 0.58 W·m^−1^·K^−1^, K_air_ = 0.024 W·m^−1^·K^−1^*^31^. The presence of CuO at Cu-Ag interface (Figure 4e) will block the heat transfer from Ag to Cu substrates since its thermal conductivity, *K_CuO_* = 20 *W·m^−1^·K^−1^*^32^, which is 20 times smaller than that of Ag, *K_Ag_ = 427 W·m^−1^·K^−1^*^31^. If the CuO coated Ag NWs are away from Cu-Ag interface, the surroundings water and air also act as a thermal barrier to keep the heat inside the Ag NWs. Therefore, one can calculate the temperature raise of one single Ag NW as indicated in Eq. 8:
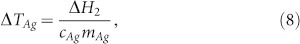
where *c* is the specific heat capacity of Ag (*c_Ag_ = 0.240 J·g^−1^·°C^−1^*)^33^ and *m_Ag_* stands for mass of Ag NW. As mentioned previously, one side of the Ag NW is 100 nm and length of Ag NW is 15 μm (Figure 3f and Figure 4e), yielding *m_Ag_ = 2.706 × 10^−12^ g*. Here, we assume the CuO layer with 10 nm thick and 100% coverage on Ag NW was completely reacted, and that, *ΔH_2_* is 0.*314 nJ*. Since *ΔH_1_* and *ΔH_3_* are excluded here, this local heat value is smaller than the calculated value of total local heat *ΔQ* using lumped capacity model. However, they are quite close. Considering the single Ag NW, *ΔT_Ag_ = 484°C*, finally (that is, temperature of Ag NW will be 504°C when room temperature is 20°C). This high temperature is striking, however one should note that this calculation is based on the assumptions of perfect surface area coverage of CuO, and that there is sufficient PVP content in the paste to support the reaction.

Furthermore, the CuO-PVP reaction in Eq. 6 was assumed as a static process and would generate heat instantaneously. However, this reaction in reality is a dynamic process with different rates of reaction depending on the concentration gradient of chemicals, temperature, pressure and the use of catalyst. Given the complexity of reaction conditions in this study, especially in the single Ag NW- Cu system as depicted in Figure 4e–f, there is no data describing their reaction rate available in the literature for a nano scale system, and it is also too difficult to be measured experimentally. Therefore, this calculated high temperature is a predicted instant maximum value. In fact, the reaction would probably last for few minutes during joining process, meaning that the heat was released gradually and accumulated in a local area because of low thermal conductivities of surroundings. Considering the long-term time scale during the actual joining process, the maximum local temperature of Ag NW would be lower than 504°C because of the limiting kinetics of the CuO-PVP reaction and heat sink effects. To generally predict the local temperature of Ag NW in this study, both the heat generation from the reaction and heat transfer into Ag NW are instantaneous as assumed previously. It suggests that the Ag NW is substantially heated above room temperature after the CuO-PVP reaction, see Figure 4f. The reaction will consume the residual PVP in the water of paste and on the surface of Ag NWs, which will remove PVP in the interfaces and make the side surface of Ag NWs exposed and activated as well for joining, similar to the end surface activation mechanism^19^. Also, this temperature rise will trigger the nanojoining of Ag NWs when they are in contact and induce a significant sintering of nanomaterials system^34^,^35^ to form networks. It is also sufficient to supply the energy for Cu and Ag atomic diffusion at Ag NW- Cu interface.

It is worth noting that this temperature is supposed to be the transient maximum value of Ag NW since the heat which would be conducted out was ignored and the reduction heat from Eq. 6 is the maximum due to the 100% coverage setup of CuO on Ag NW surface. Obviously, although this calculated temperature is a preliminary estimate, it provides some insights as to how the local heating effect contributes to joining, even though surrounding components remained near room-temperature. Our estimation shows that the negligible heat energy at bulk size cannot be ignored at a nanoscale. On the contrary, if it can be manipulated well, such weak heat energy can be collected and adopted for many new applications. It is also notable that the heat is self-generated and in-situ delivered to the interfaces at a nanoscale where the interconnections are required.

The locally generated heat mainly from Cu-to-CuO-to-Cu cycle reactions would cause atom diffusion at the Cu-Ag interfaces of Ag NWs and substrate bonding. It also conducts throughout the paste and promotes the joining of Ag NWs. The reduction of Ag NW surface due to Ag NW bonding could release energy and cause secondary local heating at Ag-Ag interface for atom interdiffusion, suggesting it is also an acceleration factor to form three dimensional Ag NW networks for interconnecting the substrates.

Another considerable effect playing positive role for joining of Ag NWs in the water-based paste is the capillary force during water evaporation process. It could bring two or more NWs into close contact with their neighboring NWs. At a nanoscale, this force can generate considerable pressure at the interface of two NWs even it is fairly weak at micro scale. In terms of pressure here, it comes from the internal system originating from the capillary force or surface tension of water rather than the external pressure applying to the system to assist the joining process. Therefore, the self-pressed process by solvent/water evaporation during joining is worth noting here. However, it is beyond the scope of this study and only local heating effects have been comprehensively studied here.

**Figure 5:**
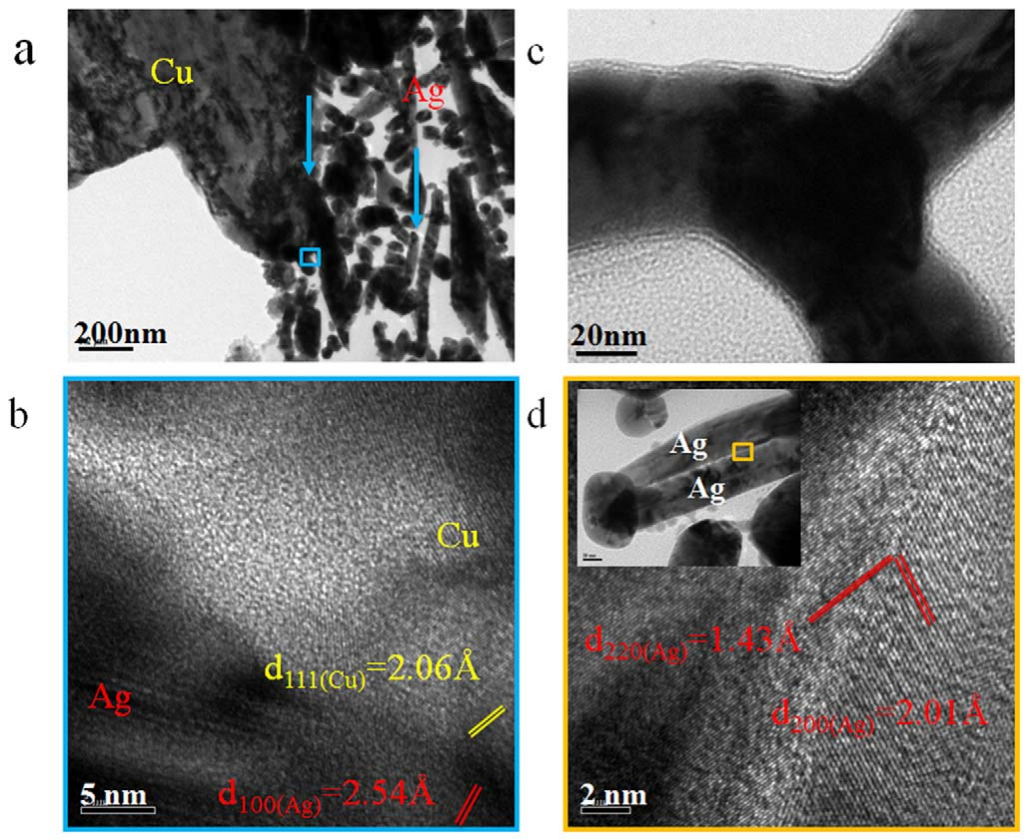
TEM images of (a) nanoporous Cu-Ag interface (arrows indicating the bonding of Ag-Cu and Ag-Ag), (b) Lattice image of Cu-Ag interface, (c) tri-junction of Ag NWs and (d) lattice image of Ag-Ag interface of joint bonded at room-temperature.

To examine if this local heating is sufficient to induce atomic diffusion and metallic bond formation, the interfaces of Cu-Ag and Ag-Ag were characterized using HRTEM. Figure 5a shows the porous structure of the interface of Cu and Ag, formed by the pressureless bonding process. The interfacial regions where the Cu surface was bonded with Ag, and Ag to Ag NW bonds formed are indicated by arrows in Figure 5a. The HRTEM image indicates that the Cu and Ag NW were metallurgically bonded, with the (100) plane of Ag and (111) of Cu connected in Figure 5b. A previous report^22^ demonstrated the connection of these planes with the orientation between Ag_111_ - Cu_200_. Ag NWs were interconnected via end-to-side, end-to-end or side-to-side manner and formed metallurgical bonds as well, and Figure 5c illustrates a tri-junction of these Ag NWs. Usually, the tips of Ag NWs have a thinner layer of PVP organics^36^ which could more readily join with others because of a smaller barrier gap and higher surface energy for atomic diffusion^19^. With the help of local heating, the sides of Ag NWs also started to connect under room temperature bonding process as shown in the inset image of Figure 5d, where (200) and (220) lattices were continuous. These observations would not be likely if the external temperature is lower than 200°C as indicated by Figure 1c. Therefore, the local heating could break down the localized organic layer on the surface of the Ag NWs and promote the interdiffusion of atoms and form the joints at even room-temperature when a Cu substrate is present.

**Figure 6:**
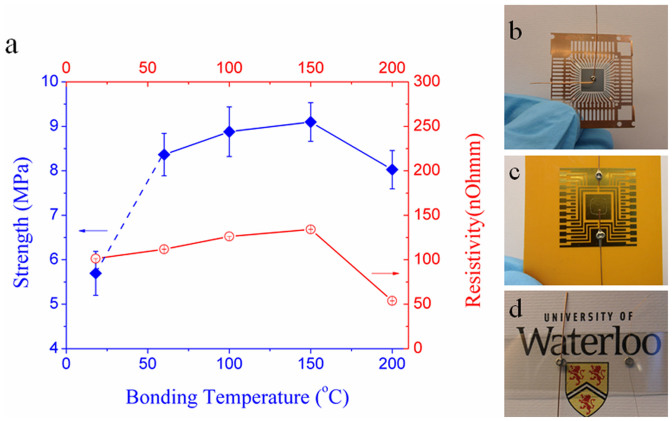
(a) Strength and resistivity of bonded Cu wire-to-wire samples using Ag NW paste. Demonstration of room-temperature pressureless bonding of Cu wire to (b) Ag plated Cu pad, (c) Au patterned polyimide and (d) transparent and flexible ITO coated PET substrates. (Reproduced from Ref. 22 by permission of The Royal Society of Chemistry).

Finally, it has been confirmed that the Ag NW paste could serve as a filler material to bond Cu substrates in different applications. The strength of a bonded sample consisting of two joined Cu wires was tested using a micro-tensile tester. The room-temperature bonded samples exhibit a strength of 5.7 MPa, while the fracture of the 250 μm Cu wires requires 13–17 MPa^37^,^38^, see Figure 6a. These failure stresses where based on the covered circumferential area in the joined wires, as discussed in prior work^38^. When the temperature was increased to 150°C, the strength increased to 9.1 MPa as a result of more intense sintering of Ag NWs and Cu-to-Ag along with a reduction of CuO nanoparticles. However, the strength reduced after 150°C due to the oxidation of Cu and decomposition of the interconnected Ag NW networks. It is worth mentioning that these results are much better than Ag and Cu NP pastes in reported in literature in the low temperature range (below 150°C)^34^,^37^.

The resistivity of the joints also follows the trend plotted in Figure 6a. The room-temperature bonded sample had a resistivity of 101.3 nΩ·m, which is on the order of the pure Cu wire. With increasing bonding and testing temperature, the resistivity increases with the temperature coefficient. At temperatures over 150°C, this decreased because of the density of filler material increasing under intense sintering. Good strength and conductivity was achieved in the Ag NW paste bonded samples at room-temperature, suggesting that it has potential applications in microelectronic industry, especially for heat-sensitive component packaging. As a demonstration of the application of the joining of Ag coated Cu chips, Au patterned polyimide and even transparent indium tin oxide coated Polyethylene terephthalate substrates have been fabricated at room-temperature, see Figure 6b–d. Furthermore, this water-based Ag NW paste can be used for printed electronic as well, because of the low sintering temperature.

In summary, the local heating effects were found for the first time in a Cu-Ag paste system when water-based silver nanowire paste was used as a filler material for flexible electronic packaging at room temperature. The reaction energy of the Cu-Ag reaction, Cu oxidation and CuO deoxidation at nanoscale close to the interfaces elevated the local temperature of Ag NW to significantly above room temperature. The lumped capacity model also confirmed that locally generated heating occurs during joining. The heat sink consisting of the Cu substrate and NW paste were close according two different calculations. Due to these local heating effects at a nanoscale, silver nanowires could form three dimensional networks via end-to-end, end-to-side and side-to-side manners and metallurgically bond with reactive assembly substrates, such as copper and silver at room temperature. These metallic bonds contributed to the excellent conductivity of 101.3 nΩ·m and joint strength of 5.7 MPa at room temperature, which could satisfy the practical demands for flexible microelectronics. This local heating was self-generated within the Cu-Ag paste system and in-situ delivered to the interfaces at a nanoscale where the interconnection to be fabricated. It is anticipated that this nanoscopic energy delivery routes opens a room for energy manipulation for temperature sensitive device manufacturing.

## Methods

### Bonding using Silver nanowire paste

Ag NWs were prepared in a polyol solution with polyvinylpyrrolidone (PVP) as a structure directing reagent using a method modified from the literature^39^,^40^. Ag NWs were washed by deionized (DI) water to remove the ethylene glycol and PVP and condensed by centrifugation. Prior to bonding, the copper wires with 0.25 mm thickness were cut into 60 mm pieces and ultrasonically cleaned in acetone for 3 minutes to remove the organics, diluted HNO_3_ for 1 minute to remove the oxide layer and rinsed in ultrapure water (electrical resistivity approximately 18 MΩ·cm). A fine needle attached on a 10 ml syringe was used to locate the Ag nanopaste between two clean copper wires. After depositing 0.05 ml high concentrating Ag nanopaste, the assembly of copper wires and paste was put at room-temperature (18°C) or heated at 60 ~ 200°C in air for 1 hour without bonding pressure.

### Testing of bonded joints

Tensile shear testing was conducted by loading the wires in the axial direction at a rate of 0.5 mm/min using a micro tensile tester (Instron 5548). Resistivity of joint, *ρ*, was calculated using *ρ = A × R/l*, where resistance (*R*) was measured by four probes low resistance Ohmmeter (DUCTER DLRO 10X), *A* and *l* are area of section and length. Field-emission scanning electron microscope (LEO 1530 FE-SEM) were used to study the microstructure of interfaces and fracture surfaces of bonded samples. Energy-dispersive X-ray spectroscopy (EDX, EDAX Pegasus 1200) was employed for elemental analysis. Interfaces of room-temperature bonded samples were characterized by observing the cross-section using transmission electron microscopy (TEM, PHILIPS CM12) and high resolution transmission electron microscopy (HRTEM, JEOL 2010F).

## Author Contributions

Y.Z. conceived the project. P.P. designed and performed the experiments. P.P., A.H., Y.L., A.G. and Y.Z. analyzed the data. P.P. wrote the manuscript. All the authors discussed the results and commented on the manuscript at all stages.

## Supplementary Material

Supplementary Information Supplementary materials

## References

[b1] Ahn J. H. *et al.* Heterogeneous three-dimensional electronics by use of printed semiconductor nanomaterials. Science 314, 1754–1757 (2006). 17170298 10.1126/science.1132394

[b2] Sun D. M. *et al.* Flexible high-performance carbon nanotube integrated circuits. Nat. Nanotechnol. 6, 156–161 (2011). 21297625 10.1038/nnano.2011.1

[b3] Someya T. *et al.* Conformable, flexible, large-area networks of pressure and thermal sensors with organic transistor active matrixes. PNAS 102, 12321–12325 (2005). 16107541 10.1073/pnas.0502392102 PMC1187825

[b4] Yang W. B. *et al.* Novel Solution Processing of High-Efficiency Earth-Abundant Cu2ZnSn(S,Se)(4) Solar Cells. Adv. Mater. 24, 6323–6329 (2012). 22969055 10.1002/adma.201201785

[b5] Ide E., Angata S., Hirose A. & Kobayashi K. F. Metal-metal bonding process using Ag metallo-organic nanoparticles. Acta Mater. 53, 2385–2393 (2005).

[b6] Li Y., Moon K. S. & Wong C. P. Electronics without lead. Science 308, 1419–1420 (2005). 15933187 10.1126/science.1110168

[b7] Peng P., Hu A. & Zhou Y. Laser sintering of silver nanoparticle thin films: microstructure and optical properties. Appl. Phys. A. 108, 685–691 (2012).

[b8] Cheng Y. T., Uang R. H., Wang Y. M., Chiou K. C. & Lee T. M. Laser annealing of gold nanoparticles thin film using photothermal effect. Microelectron. Eng. 86, 865–867 (2009).

[b9] Allen M. L. *et al.* Electrical sintering of nanoparticle structures. Nanotechnology 19, 175201 (2008). 21825662 10.1088/0957-4484/19/17/175201

[b10] Gerlich A. P. & Shibayanagi T. Liquid film formation and cracking during friction stir welding. Sci. Technol. Weld Joi. 16, 295–299 (2011).

[b11] Shah A. *et al.* Ultrasonic friction power during thermosonic Au and Cu ball bonding. J. Phys. D: Appl. Phys. 43, 325301 (2010).

[b12] Perelaer J., de Gans B. J. & Schubert U. S. Ink jet Printing and Microwave Sintering of Conductive Silver Tracks. Adv. Mater. 18, 2101–2104 (2006).

[b13] Kim C. & Forrest S. R. Fabrication of organic light-emitting devices by low-pressure cold welding. Adv. Mater. 15, 541–545 (2003).

[b14] Coughlin J. P., Williams J. J., Crawford G. A. & Chawla N. Interfacial Reactions in Model NiTi Shape Memory Alloy Fiber-Reinforced Sn Matrix “Smart” Composites. Metall. Mater. Trans. A 40A, 176–184 (2009).

[b15] Peng Y., Cullis T. & Inkson B. Bottom-up Nanoconstruction by the Welding of Individual Metallic Nanoobjects Using Nanoscale Solder. Nano Lett 9, 91–96 (2009). 19072096 10.1021/nl8025339

[b16] Garnett E. C. *et al.* Self-limited plasmonic welding of silver nanowire junctions. Nat. Mater. 11, 241–249 (2012). 22306769 10.1038/nmat3238

[b17] Zhou Y. Microjoining and Nanojoining. (Woodhead, England; 2008).

[b18] Lu Y., Huang J. Y., Wang C., Sun S. H. & Lou J. Cold welding of ultrathin gold nanowires. Nat. Nanotechnol. 5, 218–224 (2010). 20154688 10.1038/nnano.2010.4

[b19] Peng P., Liu L., Gerlich A. P., Hu A. & Zhou Y. N. Self-Oriented Nanojoining of Silver Nanowires via Surface Selective Activation. Part. Part. Syst. Charact. 30, 420–426 (2013).

[b20] Wakuda D., Kim K.-S. & Suganuma K. Room temperature sintering of Ag nanoparticles by drying solvent. Scr. Mater. 59, 649–652 (2008).

[b21] Magdassi S., Grouchko M., Berezin O. & Kamyshny A. Triggering the sintering of silver nanoparticles at room temperature. ACS Nano 4, 1943–1948 (2010). 20373743 10.1021/nn901868t

[b22] Peng P. *et al.* Room-temperature pressureless bonding with silver nanowire paste: towards organic electronic and heat-sensitive functional devices packaging. J. Mater. Chem. 22, 12997–13001 (2012).

[b23] Peng P., Huang H., Hu A. M., Gerlich A. P. & Zhou Y. N. Functionalization of silver nanowire surfaces with copper oxide for surface-enhanced Raman spectroscopic bio-sensing. J. Mater. Chem. 22, 15495–15499 (2012).

[b24] Lienhard J. H. A heat transfer textbook. (Courier Dover Publications, 2011).

[b25] Engineering Toolbox- *Heat transfer coefficient*. Availale at: http://www.engineeringtoolbox.com/overall-heat-transfer-coefficient-d_434.html (Accessed: 1^st^ May 2013).

[b26] Hoppe C. E., Lazzari M., Pardinas-Blanco I. & Lopez-Quintela M. A. One-step synthesis of gold and silver hydrosols using poly(N-vinyl-2-pyrrolidone) as a reducing agent. Langmuir 22, 7027–7034 (2006). 16863256 10.1021/la060885d

[b27] Xiong Y. J. *et al.* Poly(vinyl pyrrolidone): A dual functional reductant and stabilizer for the facile synthesis of noble metal nanoplates in aqueous solutions. Langmuir 22, 8563–8570 (2006). 16981776 10.1021/la061323x

[b28] Zhan Y. J., Lu Y., Peng C. & Lou J. Solvothermal synthesis and mechanical characterization of single crystalline copper nanorings. J. Cryst. Growth 325, 76–80 (2011).

[b29] Zhang J.-M., Ma F. & Xu K.-W. Calculation of the surface energy of FCC metals with modified embedded-atom method. Appl. Surf. Sci. 229, 34–42 (2004).

[b30] Barin I., Sauert F., Schultze-Rhonhof E. & Sheng W. S. Thermochemical data of pure substances, Vol. 6940. (VCH Weinheim, Germany 1993).

[b31] Beardmore R. Heat transfer. Availale at: http://www.roymech.co.uk/Related/Thermos/Thermos_HeatTransfer.html (Accessed: 1^st^ May 2013).

[b32] Kwak K. & Kim C. Viscosity and thermal conductivity of copper oxide nanofluid dispersed in ethylene glycol. Korea-Australia Rheology Journal 17, 35–40 (2005).

[b33] Stretton T. Tom Stretton's Chemistry Pages-*Specific heat capacity table* (2014). Availale at: http://www2.ucdsb.on.ca/tiss/stretton/database/Specific_Heat_Capacity_Table.html (Accessed: 1^st^ May 2013).

[b34] Hu A. *et al.* Low temperature sintering of Ag nanoparticles for flexible electronics packaging. Appl. Phys. Lett. 97, 153117 (2010).

[b35] Ngoc H. N., Hu A. M., Persic J. & Wen J. Z. Molecular dynamics simulation of energetic aluminum/palladium core-shell nanoparticles. Chem. Phys. Lett. 503, 112–117 (2011).

[b36] Sun Y. G., Mayers B., Herricks T. & Xia Y. N. Polyol synthesis of uniform silver nanowires: A plausible growth mechanism and the supporting evidence. Nano Lett. 3, 955–960 (2003).

[b37] Yan J. F., Zou G. S., Hu A. M. & Zhou Y. N. Preparation of PVP coated Cu NPs and the application for low-temperature bonding. J. Mater. Chem. 21, 15981–15986 (2011).

[b38] Peng P., Hu A. M., Zhao B. X., Gerlich A. P. & Zhou Y. N. Reinforcement of Ag nanoparticle paste with nanowires for low temperature pressureless bonding. J. Mater. Sci. 47, 6801–6811 (2012).

[b39] Sun Y. G., Gates B., Mayers B. & Xia Y. N. Crystalline silver nanowires by soft solution processing. Nano Lett. 2, 165–168 (2002).

[b40] Sun Y. G. & Xia Y. N. Large-scale synthesis of uniform silver nanowires through a soft, self-seeding, polyol process. Adv. Mater. 14, 833–837 (2002).

