# Supplementary material for: Self-generated Local Heating Induced Nanojoining for Room Temperature Pressureless Flexible Electronic Packaging
Source: Sci Rep. 2015 Mar 19;5:9282. doi: 10.1038/srep09282 (PMC4365387; doi:10.1038/srep09282)
Supplement: Supplementary Information — Supplementary materials [file srep09282-s1.pdf]

# Self-generated Local Heating Induced Nanojoining for Room Temperature Pressureless Flexible Electronic Packaging

*Peng Peng, Anming Hu, Adrian P. Gerlich, Yangai Liu, Y. Norman Zhou*

## Supplementary information

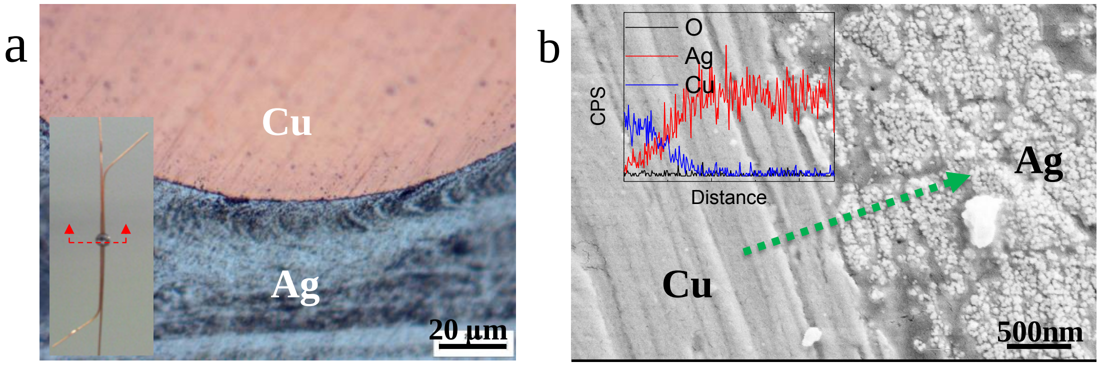


**Figure S 1**. (a) Optical images of cross-section of Ag NW bonded sample at room temperature, with the bonded wires shown in the inset; (b) SEM of cross-sectioned interface with EDX line scan.

**Supplementary Note 1, the formation of CuO NPs on Ag NWs.** With water-based paste exposure to atmosphere, Cu ions will be formed by oxidizing the surface of Cu substrates. This electrochemical reaction is facilitated by different chemical potentials of Cu and Ag. These Cu ions migrate to the surface of Ag NWs, and combine with O ions originated from soluble $O_2$ to form CuO NPs. The EDX results show that the composition is Cu and O, see Figure S 2a (Mo grid was used for TEM observation). Meanwhile, the micro-XRD pattern indicates the formation of CuO on Cu substrate, as shown in Figure S 2b. The characteristic peaks at 35$^o$ ($2\theta$) and 62$^o$ ($2\theta$) were weak because the CuO amount is fairly low. The low crystallinity of CuO NPs (possibly amorphous) can also lower the peaks.

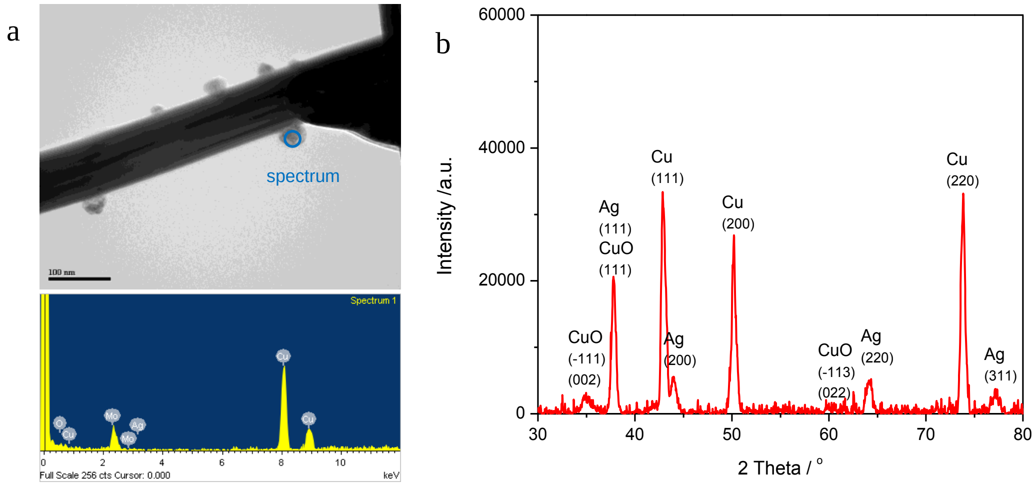


Figure S 2 (a) EDX characterization of NPs on Ag NW surface. (b) Micro-XRD results of NPs and Ag NWs on a Cu substrate.

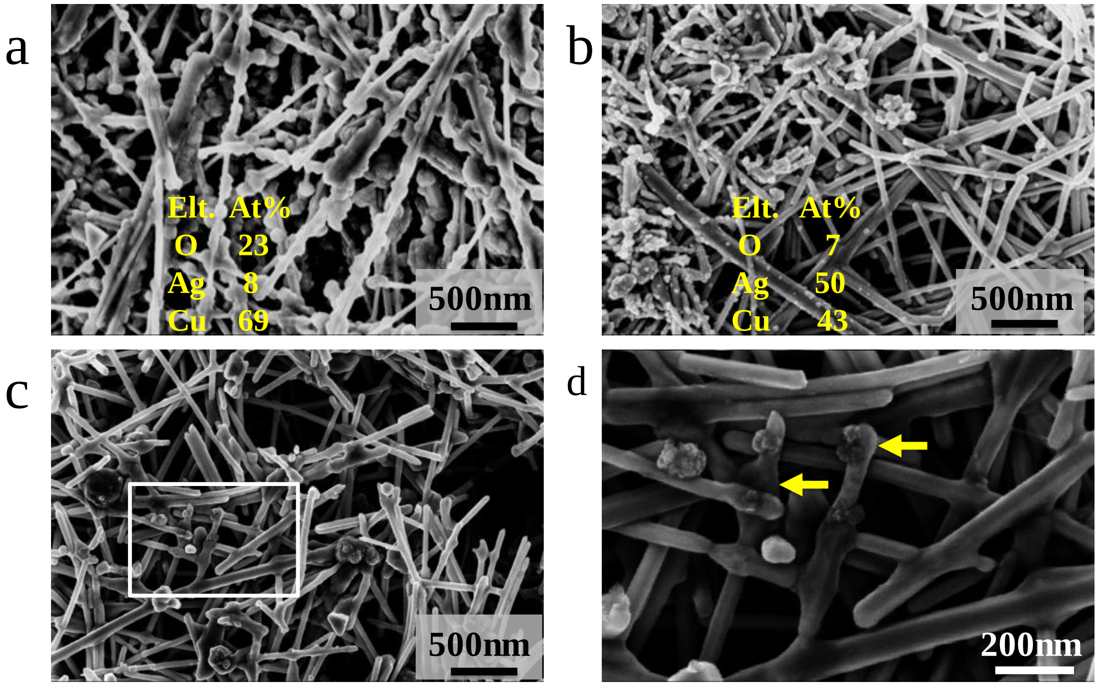


**Figure S 3**. SEM micrographs of fractured interfaces of room-temperature bonded samples: (a) without heating process, (b) heated at 100°C and (c) 150°C for 1 hr and its high resolution image (d). With the heating temperature increasing, the CuO nanoparticles on Ag NW surfaces decreased.

**Supplementary Note 2, the reduction of CuO by PVP.** PVP was used not only as a stabilizer but also a reducing agent to synthesis noble metal nanomaterials.[1-3] The reaction could be drawn as **Scheme 1**. According to the TEM observation, the reduced Cu NPs coated on Ag NW surface as illustrated in Figure S 4a. EDX indicates that the composition of this layer was Cu without O (Mo was from the grid). Also, after the reaction with PVP solution at 80°C, no CuO has been detected using micro-XRD, as shown in Figure S 4b. This confirmed the reduction of CuO by PVP. After the reduction, Cu NPs possess a better crystallinity than that of CuO NPs. This increases the intensity of Cu peaks.

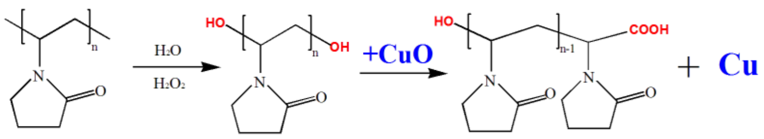


Scheme 1 The chemical reaction of CuO with PVP.

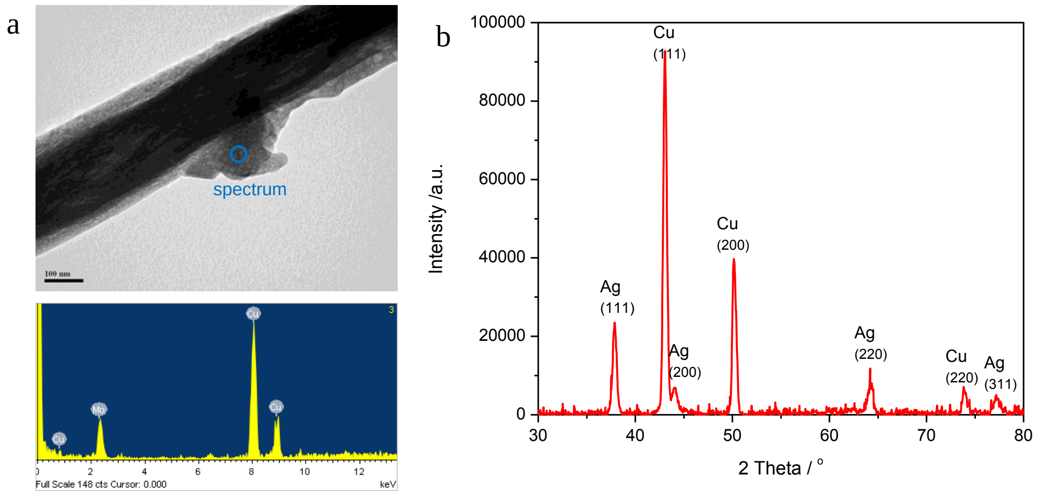


Figure S 4 (a) EDX results on NP and NW after reaction with PVP solution at 80°C. (b) Micro-XRD results of NPs and Ag NWs on Cu substrate after reaction with PVP solution at 80°C.

1. Zhan, Y.J., Lu, Y., Peng, C. & Lou, J. Solvothermal synthesis and mechanical characterization of single crystalline copper nanorings. *J. Cryst. Growth* **325**, 76-80 (2011).
2. Xiong, Y.J. et al. Poly(vinyl pyrrolidone): A dual functional reductant and stabilizer for the facile synthesis of noble metal nanoplates in aqueous solutions. *Langmuir* **22**, 8563-8570 (2006).
3. Hoppe, C.E., Lazzari, M., Pardinas-Blanco, I. & Lopez-Quintela, M.A. One-step synthesis of gold and silver hydrosols using poly(N-vinyl-2-pyrrolidone) as a reducing agent. *Langmuir* **22**, 7027-7034 (2006).
